# A Ratiometric Sensor for Imaging Insulin Secretion in Single β Cells

**DOI:** 10.1016/j.chembiol.2017.03.001

**Published:** 2017-04-20

**Authors:** Martina Schifferer, Dmytro A. Yushchenko, Frank Stein, Andrey Bolbat, Carsten Schultz

**Affiliations:** 1 Interdisciplinary Chemistry Group, Cell Biology & Biophysics Unit, European Molecular Biology Laboratory (EMBL), Meyerhofstrasse 1, 69117 Heidelberg, Germany; 2 Group of Chemical Biology, Institute of Organic Chemistry and Biochemistry, Academy of Sciences of the Czech Republic, Flemingovo namesti 2, 16610 Prague 6, Czech Republic; 3 Department of Physiology and Pharmacology, Oregon Health and Science University, Portland, OR 97237, USA

**Keywords:** granule, insulin, biosensor, fluorescence, TIRF, calcium, oscillation, tolbutamide, potassium channel, glucose, superfolder GFP, mCherry

## Abstract

Despite the urgent need for assays to visualize insulin secretion there is to date no reliable method available for measuring insulin release from single cells. To address this need, we developed a genetically encoded reporter termed RINS1 based on proinsulin superfolder GFP (sfGFP) and mCherry fusions for monitoring insulin secretion. RINS1 expression in MIN6 β cells resulted in proper processing yielding single-labeled insulin species. Unexpectedly, glucose or drug stimulation of insulin secretion in β cells led to the preferential release of the insulin-sfGFP construct, while the mCherry-fused C-peptide remained trapped in exocytic granules. This physical separation was used to monitor glucose-stimulated insulin secretion ratiometrically by total internal reflection fluorescence microscopy in single MIN6 and primary mouse β cells. Further, RINS1 enabled parallel monitoring of pulsatile insulin release in tolbutamide-treated β cells, demonstrating the potential of RINS1 for investigations of antidiabetic drug candidates at the single-cell level.

## Highlights

• Genetically encoded proinsulin-based reporter RINS1 for sensing insulin secretion
• RINS1 enables ratiometric monitoring of stimulated exocytosis in single β cells
• RINS1 enables visualization of drug-mediated modulation of insulin secretion
• RINS1 can be combined with other fluorescent sensors in cultured and primary cells

## Graphical Abstract

**Figure:**
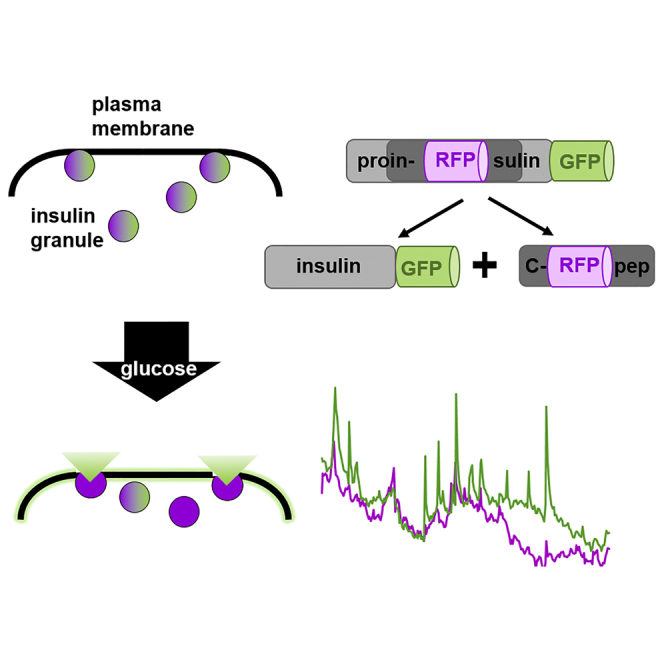

## Teaser

Schifferer et al. describe a genetically encoded reporter termed RINS1 for ratiometric sensing of insulin secretion. RINS1 is based on proinsulin-sfGFP and -mCherry fusions and enables monitoring of insulin dynamics upon drug treatment at different subcellular sites within β cells.

## Introduction

Diabetes is one of the most common diseases worldwide. It manifests itself by a faulty regulation of blood sugar by insulin. There are two common types of diabetes: type 1 and type 2 diabetes. Type 1 diabetes is characterized by the autoimmune destruction and drastic loss of insulin-secreting pancreatic β cells leading to hyperglycemia (Fu et al., 2013). The most common treatment for type 1 diabetes with usually little residual insulin secretion is the subcutaneous injection of recombinant human insulin before or after food intake. Type 2 diabetes on the other hand is the more common type of diabetes (representing 90% of diabetic cases worldwide) and is characterized by insulin resistance, often in combination with reduced insulin secretion. Many less-severe cases of type 2 do not require insulin substitution but the use of drugs that stimulate insulin secretion such as metformin, tolbutamide, or others (Rorsman, 2005). In an experimental setup, insulin secretion is usually determined by an ELISA assay which of course is limited to detection of bulk insulin released by an entire pancreas, a group of islets, or cultured cells. At the single-cell level, patch-clamp measurements are quite common (Guo et al., 2014, Ammala et al., 1991). Surprisingly, there are only a few single-cell-based fluorescent assays available to directly monitor the fusion of the secretory granules and the release of insulin. A variety of fluorescent protein (FP)-tagged constructs has been developed to monitor exocytosis from β cells. For example, single-cell imaging of granules was first achieved by expressing a chimera of the dense-core secretory granule membrane glycoprotein phogrin and EGFP (Pouli et al., 1998), which was later combined with the application of the small dye acridine orange to image exocytosis from β cells (Tsuboi et al., 2000). There are also approaches based on monitoring release of other molecules which are concomitantly secreted with insulin such as Neuropeptide Y (Ohara-Imaizumi et al., 2002, Ohara-Imaizumi et al., 2007), tissue plasminogen activator (Tsuboi et al., 2004), or zinc ions (Li et al., 2011, Pancholi et al., 2014, Lemaire et al., 2009) by confocal and total internal reflection fluorescence (TIRF) microscopy. This work is nicely summarized in Rutter (2004) and Loder et al. (2013). Insulin secretion is mainly stimulated by strong intracellular calcium oscillations (Soria and Martin, 1998). Accordingly, calcium-sensitive indicators, but also probes that measure changes in pH, are employed. While enormously useful to better understand the underlying signaling network, such tools often monitor vesicle fusion of any kind and not just insulin-filled granule fusion. Typical strategies for direct visualization of insulin secretion involve simple FP tagging of the insulin C terminus (Ohara-Imaizumi et al., 2002, Ohara-Imaizumi et al., 2004, Ohara-Imaizumi et al., 2007) or insertion of an FP into the C-peptide (Michael et al., 2004, Michael et al., 2006, Watkins et al., 2002, Michael et al., 2004, Burns et al., 2015). As an alternative, fusion protein tags that bind fluorescent dyes are available allowing for pulse-chase labeling (Ivanova et al., 2013, Hoboth et al., 2015). However, the non-ratiometric datasets are very difficult to interpret. Ideally, one would exclusively image the fusion of single, insulin-filled secretory granules with the plasma membrane and the corresponding hormone release ratiometrically in real time and with high spatial resolution.

## Results

### Insulin Reporter Design

**Figure 1:**
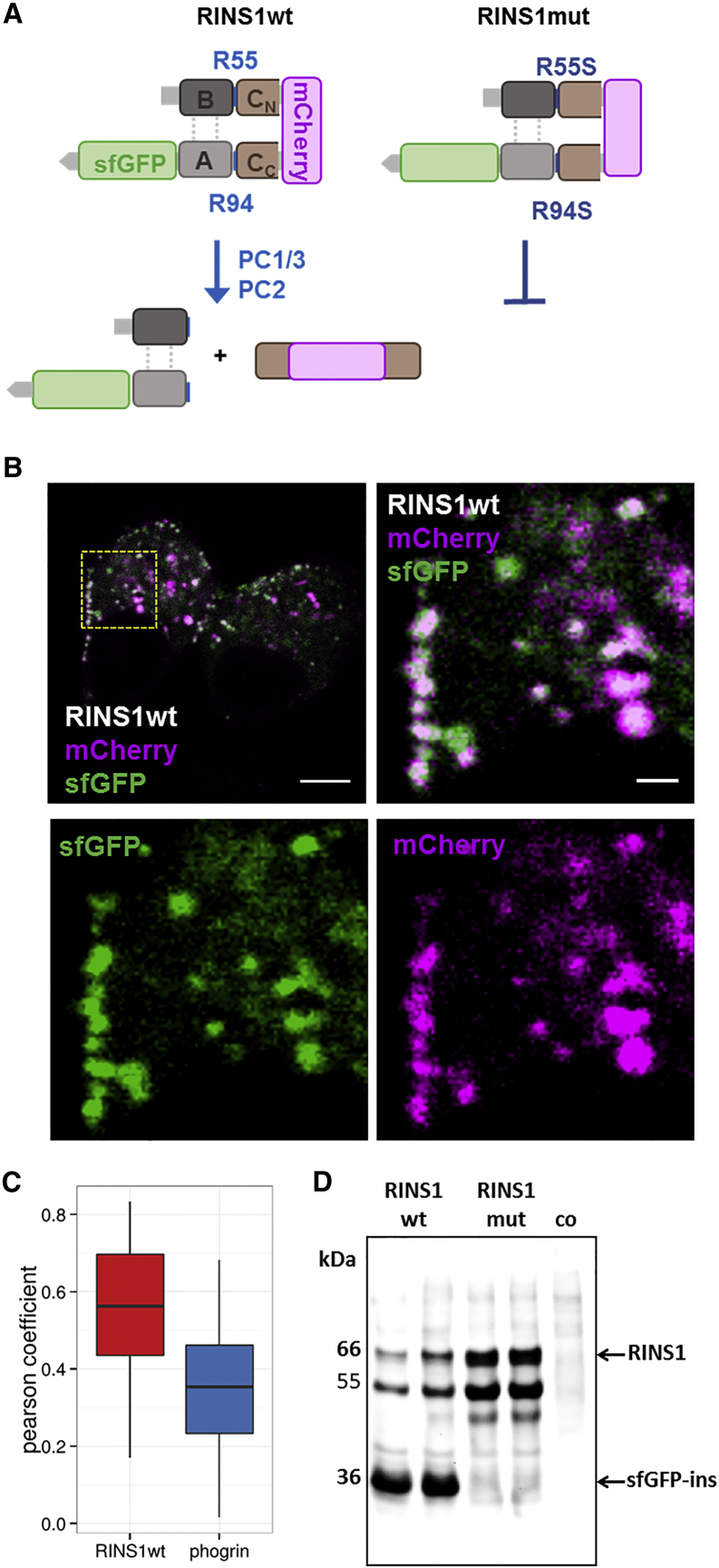
Proinsulin-Based RINS1 Sensor Design and Expression in β Cells (A) Cartoon of RINS1 and of its non-cleavable mutant RINS1mut. (B) Confocal images of MIN6 β cells expressing RINS1. Enlargement of the selected square (top right). Merged images (top), single channels (bottom), mCherry (magenta), sfGFP (green). Scale bar, 5 μm (top left), 1 μm (top right). See also Figure S1. (C) Quantification (box and whisker plot) of the colocalization of RINS1 mCherry-only with granule marker phogrin (blue, n = 168) and sfGFP in RINS1 (red, n = 164) by Pearson coefficient. (D) Western analysis of MIN6 lysates using an anti-insulin antibody. Cells were transfected by RINS1 or mut, non-transfected cells served as controls. Anti-insulin western blotting showed bands for proinsulin RINS1 (66 kDa), sfGFP-insulin (36 kDa).

Here we report a ratiometric sensor for insulin secretion based on a double-fluorescent-fusion construct of proinsulin. The sensor is cleaved during secretory granule maturation by specific proteases (called convertases) to form the physiologically active A-B chains of insulin bridged by disulfide bonds and the inactive C-peptide (Figure 1A) (Davidson, 2004). Triggered by elevated glucose levels and increases in intracellular calcium levels, the mature granules fuse with the plasma membrane, in the first minutes predominantly in a kiss-and-run fashion (Tsuboi and Rutter, 2003), to release their content including the quasi-crystalline insulin (Nicolson et al., 2009). Therefore, the loss of insulin would be one of the most direct methods of monitoring insulin secretion, provided that the signal is ratiometric rather than a drop in fluorescence.

To measure ratiometrically, we fused both the C-peptide and the A-peptide to the pH-insensitive mCherry and superfolder GFP (sfGFP), respectively (Figure 1A). Efficient tagging at the chosen target site (between proline 72 and glycine 73) was reported previously for the insertion of Emerald GFP (Michael et al., 2006). sfGFP was fused to the C terminus of the insulin A chain using a short flexible GGA linker. The sensor was termed RINS1 (Figure 1A). We took advantage of the improved folding kinetics of sfGFP over EGFP (Ohara-Imaizumi et al., 2002, Ohara-Imaizumi et al., 2004, Ohara-Imaizumi et al., 2007), especially in the endoplasmic reticulum (Aronson et al., 2011) to ensure that early insulin species were readily visible.

### RINS1 Expression and Processing in MIN6 β Cells

**Figure 2:**
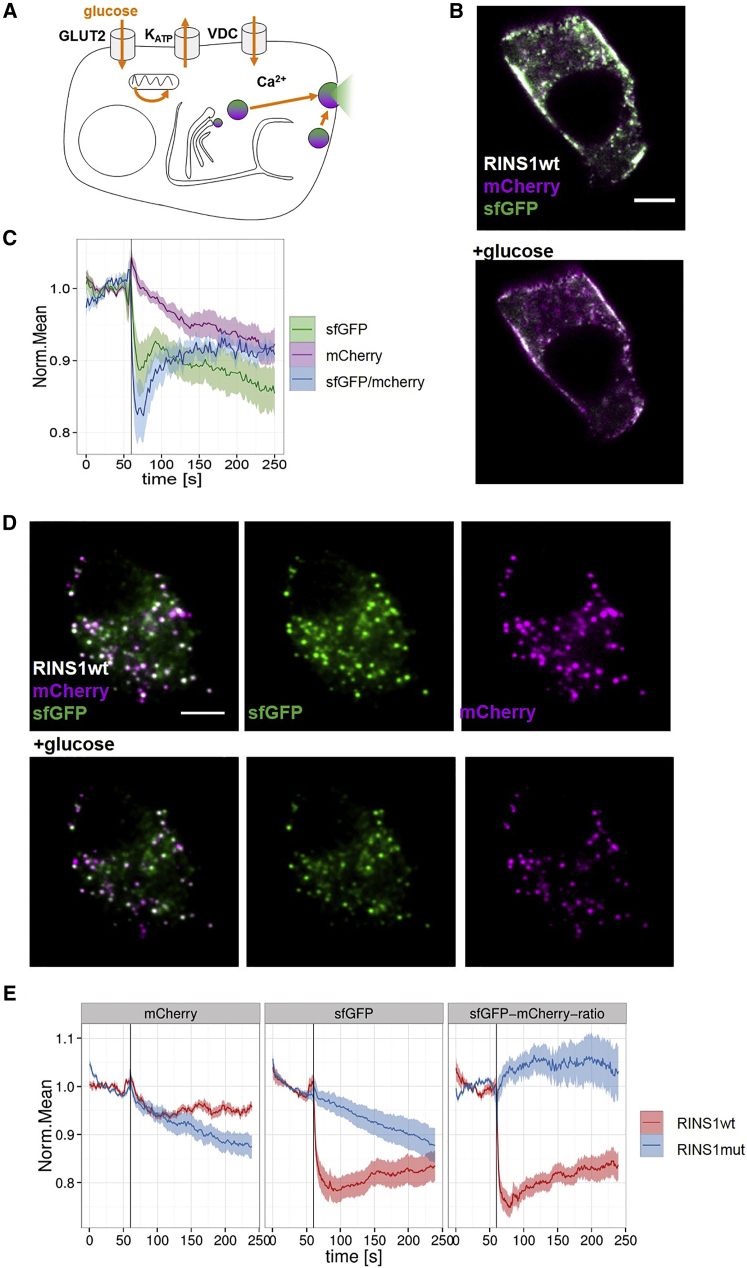
Monitoring Stimulated Insulin Secretion in Single β Cells (A) Cartoon demonstrating glucose-stimulated secretion in β cells expressing RINS1. SG, secretory granule; GLUT2, glucose transporter 2; KATP, ATP-sensitive potassium-channel; VDC, voltage-dependent channel. (B–E) MIN6 cell-expressing RINS1. (B) Confocal microscopy of MIN6 before (top) and after (bottom) addition of 20 mM glucose when sfGFP-tagged insulin (green) released from the cell while C-peptide-mCherry (magenta) stayed in the granules. Scale bar, 5 μm. (C) Whole-field of view quantification of RINS1 sfGFP, mCherry, and sfGFP/mCherry ratio channels observed over time by confocal microscopy. sfGFP (green), mCherry (magenta), and sfGFP/mCherry (blue). n = 19. (D) TIRF imaging of RINS1-expressing MIN6 cells before (top) and after (bottom) addition of 20 mM glucose. See also Figure S2. (E) Quantification of TIRF imaging data of MIN6 cells expressing RINS1 (red, n = 20) and RINS1mut (blue, n = 16) stimulated after 60 s with 20 mM glucose (black line). Traces for sfGFP, mCherry, and sfGFP/mCherry ratio channels are shown. (C, E) Error bars represent standard error of the mean.

RINS1 was transiently expressed in cultured mouse MIN6 β cells (passage 25–35) (Nakashima et al., 2009, Ishihara et al., 1993, Miyazaki et al., 1990, Cheng et al., 2012) (Figure 2B), as well as in primary β cells (Figure S8) under control of a cytomegalovirus promoter. RINS1-positive cells showed a granular signal of both FPs imaged by confocal microscopy (Figure 1B) with a subgroup of granules aligning at the plasma membrane and others located within the cytosol. Efficiency of MIN6 cell transfection with RINS1-containing plasmid in the presence of Lipofectamine 2000 was about 50% within an islet-like assembly (Figure S1). For transfection of primary β cells we used an adenoviral vector that led to about 80% of transduction. To control for proteolytic cleavage, we constructed a mutant with disabled protease cleavage sites between the A-B- and C-peptides (called RINS1mut). PFA-fixed mouse MIN6 β cells expressing RINS1 showed significant overlap (Pearson coefficient 0.56 ± 0.17, Figure 1C) of sfGFP and mCherry fluorescence, indicating that the two fluorophores were located in the same vesicles in most cases (Figure 1B). Moreover, by applying high-precision correlative fluorescence and electron microscopy, we demonstrated that MIN6 cells expressing RINS1 have very similar granule morphology to non-transfected cells (Figure S10). They contain characteristic subcellular structures in electron microscopy images that likely correspond to insulin crystals (Figure S10C). Co-expressing the granule marker EGFP-phogrin with an RINS1 construct without the sfGFP fusion (RINS1_mCherryonly_, Figure S2B) confirmed its identity as a secretory granule cargo (Pearson coefficient 0.35 ± 0.16, Figure 1C). Cell lysate analysis by western blotting using anti-insulin antibody demonstrated that RINS1wt was readily hydrolyzed with a ratio of non-processed proinsulin fused to both FPs (66 kDa) to mature insulin-sfGFP (36 kDa) of 0.09 (Figure 1D). Application of anti-GFP antibody confirmed RINS cleavage (Figure S13), but led to a different ratio mainly due to the low specificity of the anti-GFP antibody for GFP over mCherry (Figure S14). Interestingly, the western blot with anti-mCherry antibody demonstrated that C-peptide-mCherry generated from RINS1 existed predominantly in a dimeric form (Figure S13). In lysates of the cells expressing RINS1mut no mature insulin-sfGFP fusion construct was detected due to the lack of convertase cleavage (Figure 1D).

### Ratiometric Imaging of Stimulated Insulin Secretion

To study the fate of RINS1 during secretion, we stimulated cells by addition of 20 mM glucose and observed secretory granules by confocal microscopy (Figure 2). Stimulated insulin secretion induced a rapid decrease in RINS1 sfGFP emission (−20%) but not the mCherry signal (Figure 2C), indicating that insulin-sfGFP was released while mCherry-C-peptide was retained in the cells. The difference in the secretion levels of insulin-sfGFP and mCherry-C-peptide was further confirmed by performing fluorescence characterization of the material released into the extracellular medium by MIN6 cells transiently expressing RINS1 (Figure S11). It showed a stronger increase in the levels of sfGFP fluorescence compared with mCherry levels (Table S1). Consequently, glucose-induced insulin secretion could be monitored at the single-cell level by fluorescence microscopy as a decrease in intracellular sfGFP/mCherry ratio as well as at the cell-batch level by applying fluorescence spectroscopy as an increase of sfGFP/mCherry ratio in the culture medium. As the change in exocytotic vesicle pH during kiss-and-run secretion may affect the sfGFP/mCherry emission ratio, we also tested for the pH dependence of the two fluorophores by addition of NH_4_Cl to MIN6 cells. Our result excludes a contribution of pH to the observed decrease of the sfGFP/mCherry ratio during secretion (Figure S12). We did not observe any significant contribution of FRET from sfGFP to mCherry (Figure S2). There was only a slightly higher FRET signal in RINS1mut compared with RINS1 granules. This is explained by the higher probability for intramolecular compared with intermolecular FRET as the RINS1mut reporter fluorophores are not separated by the protein convertases.

We then performed selective imaging of plasma membrane-localized secretory granules in RINS1-expressing MIN6 cells taking advantage of TIRF microscopy (Figure S3). RINS1 showed a fast decrease in sfGFP emission after stimulation by glucose (Figures 2D and 2E). To control for the difference of mCherry and sfGFP protein characteristics, we tested RINS1mut-expressing MIN6 cells and found no major difference in their emission after stimulation (Figure 2E). The amplitude of RINS1 response to glucose stimulation is independent of its expression levels or the pre-stimulation sfGFP/mCherry ratio (Figure S4). We also stimulated cells with KCl (30 mM), which led to direct depolarization and subsequent calcium influx independent of glucose levels. We found a faster recovery of the sfGFP/mCherry ratio after stimulation with KCl compared with glucose stimulation in MIN6 cells (Figure S5), but slower recovery of the sfGFP/mCherry ratio after stimulation with KCl in primary β cells (Figure S9).

### Visualization of Insulin Transients and Calcium in Tolbutamide-Treated β Cells

**Figure 3:**
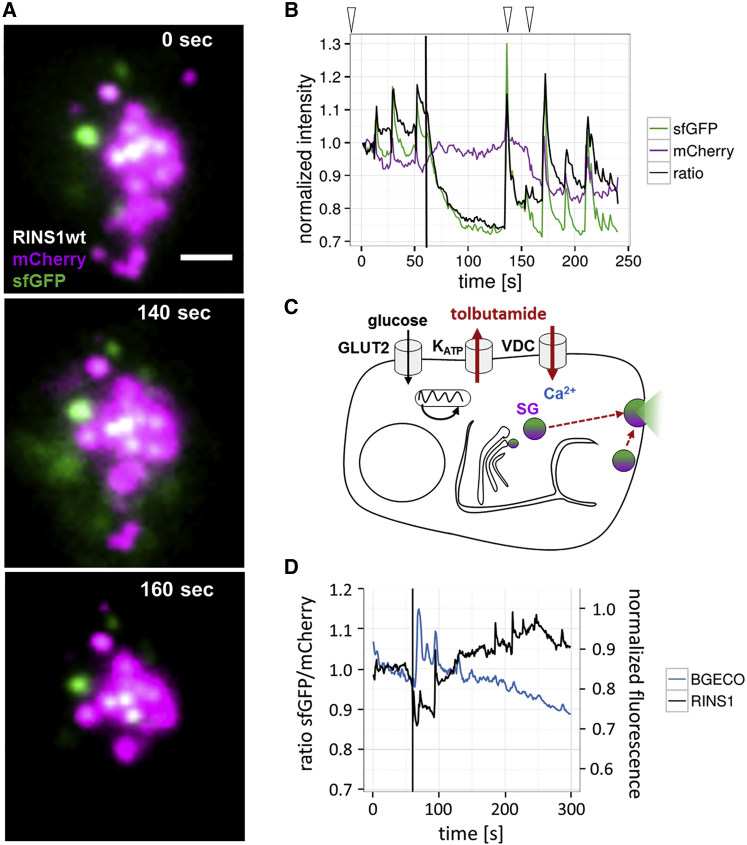
Drug-Mediated Modulation of Insulin Secretion Visualized by RINS1 in MIN6 Cells Imaged by TIRFM (A and B) The effect of tolbutamide incubation (100 μM, 30 min) on insulin secretion at the single-cell level. (A) Example TIRF images of RINS1-bearing granules in a single β cell at defined time points. For a full movie, see the Supplemental Information. Scale bar, 2 μm. (B) sfGFP (green), mCherry (magenta) intensity, and ratio sfGFP/mCherry (blue) changes over time. Arrow heads indicate time points of images in (A). (C) Schematic drawing of sulfonylurea drugs affecting insulin secretion. Tolbutamide (red) inhibits K_ATP_ channels, leading to depolarization and calcium influx even in the absence of glucose. (D) MIN6 cell-expressing RINS1 and B-GECO treated with tolbutamide (100 μM, 30 min) and stimulated by glucose after 60 s. B-GECO (blue) indicating calcium levels and RINS1 sfGFP/mCherry ratio (black) of for a single cell is plotted.

RINS1 has proven useful to monitor physiological changes in insulin secretion and therefore has the potential to monitor drug effects on secretion dynamics. We therefore treated RINS1-expressing MIN6 cells with the sulfonylurea drug tolbutamide, a potent inhibitor of ATP-driven potassium channels in β cells (Mariot et al., 1998), and monitored insulin release by TIRF microscopy. Inhibition of potassium channels led to cell depolarization, calcium influx, and increased insulin secretion (Figure 3). After addition of glucose, a general drop in the sfGFP channel was observed, similarly to previous experiments with no drug treatment, while the red channel fluorescence persisted (Figures 3A and 3B). Surprisingly, we registered additional spontaneous insulin transients (Figure 3B). We frequently observed a bright increase in both wavelengths, albeit much stronger in the green channel, probably when groups of vesicles opened. Each event appears as a bright release in real-time movies (see Movie S2) and are probably visible because the FPs are released into the small space between cell and coverslip in the slim focal plane generated by TIRF illumination. Such insulin spikes were not observed in the absence of sulfonylurea drugs. The result is consistent with the known activation of first-phase secretion by tolbutamide (Eliasson et al., 2003). To dissect the calcium response from the actual insulin release (Figure 3C), we co-expressed the calcium sensor B-GECO1 and RINS1 (Figure 3D) in MIN6 cells. Upon tolbutamide treatment and glucose stimulation, we observed insulin spikes that coincided with increases in calcium oscillation amplitudes only at early time points. Such spontaneous release might be either due to calcium-dependent and -independent insulin secretion (Ammala et al., 1993, Sakuma et al., 1995) or the loss of exocytosis-competent granules and hence demonstrates the relevance of an insulin-specific reporter.

## Discussion

RINS1 is a simple double-labeled proinsulin fusion that seems to be processed similarly to wild-type proinsulin. In fact, single-labeled proinsulin-sfGFP and -mCherry fusions behave similarly (Figure S2). Upon granule maturation, insulin and the C-peptide separate. Unexpectedly, only the insulin construct was secreted while the C-peptide-mCherry fusion remained in the recycling vesicle. This is especially surprising taking into account that C-peptide levels are routinely used to determine residual insulin secretion in the blood of patients who are treated with recombinant insulin (just the A-B chain). Hence, endogenous C-peptide secretion levels must be substantial. It appears that mCherry extension prevents C-peptide secretion. One of the possible explanations is the dimerization of C-peptide-mCherry (illustrated in Figure S13), which decreases the propensity of the C-peptide-mCherry fusion for secretion. Alternatively, it might be due to the fact that fully processed insulin crystallizes in the secretory granules and has likely less contact to other structures within the granule compared with C-peptide.

In any case, only the physical detachment of sfGFP-insulin and C-peptide-mCherry made the ratiometric readout possible. This single-cell secretion assay will help to investigate the signaling network in β cells because it provides a physiologically meaningful readout when cells are stimulated or inhibited and will also be instrumental in combination with other fluorescent reporters that monitor signaling events. Especially the correlation with other signaling or secretory events in the same cell will open new ways for studying insulin secretion and its regulation. In the future, this single-cell technique for insulin secretion will be very useful for probing the effect of drugs and drug candidates modulating the calcium and potassium levels of β cells.

## Significance

**Measuring insulin levels and secretion is common practice in the lab and in clinics. However, there is a lack of suitable single-cell methods for monitoring the β cell signaling network. So far, mostly indirect readouts such as calcium levels or granule co-cargo secretion were monitored. Here we describe a novel method for direct proinsulin tagging by two fluorescent proteins at distinct sites in mouse β cells. This allows, for the first time, the microscopic distinction of different insulin species. We show that sfGFP-labeled mature insulin is preferentially released while mCherry-C-peptide is retained in the insulin granule. Consequently, RINS1 enables ratiometric imaging of stimulated insulin secretion and the monitoring of insulin dynamics upon drug treatment. For the first time, we show insulin transients at different subcellular sites within β cells. We demonstrate that RINS1 can be combined with calcium detection in single cells. This opens the unique opportunity of applying RINS1 to β cells together with other fluorescent sensors. The direct correlation of several signals in the same cell will be extremely useful for understanding the mechanisms of insulin secretion, both at the level of the driving signaling network as well as for unraveling the complex machinery that drives the exocytosis of the secretory granules. Such experiments are much more useful at the single-cell level due to the large cell-to-cell variability of cultured and primary cells. Finally, RINS1 will help to dissect the effects of diabetes-relevant drug candidates on different signaling pathways in β cells. Genetic encoding of this novel reporter will enable its application in diabetic model systems with clinical relevance and viral transfection will permit working with primary β cells from donors.**

## STAR★Methods

### Key Resources Table

**Table:** 

REAGENT or RESOURCE	SOURCE	IDENTIFIER
**Antibodies**		

Rabbit polyclonal primary anti-insulin (unconjugated)	SantaCruzBiotech	Sc-9168; RRID: AB_2126540
Rabbit polyclonal primary anti-GFP (unconjugated)	Molecular Probes<	A11122; RRID: AB_221569
Rabbit monoclonal primary anti-mCherry (unconjugated)	Molecular Probes	M11217; RRID: AB_2536611
Goat polyclonal secondary anti-rabbit (HRP conjugated)	Invitrogen	31460; RRID: AB_228341
Goat polyclonal secondary anti-rat (HRP conjugated)	Invitrogen	31470; RRID: AB_228356

**Bacterial and Virus Strains**		

RINS 1adenoviral vector	Vector Biolabs	Type 5, dE1/E3

**Biological Samples**		

Mouse pancreatic islets	EMBL animal facility	C57BL6 mice

**Chemicals, Peptides, and Recombinant Proteins**		

Tolbutamide	Sigma	T0891
Histopaque®-1083	Sigma	10831
Histopaque®-1119	Sigma	11191
Collagenase NB8	SERVA	17456
Trypsin	Sigma	T4424

**Experimental Models: Cell Lines**		

Mouse MIN6 cell line, passage number 25-35	Miyazaki laboratory, Oaka University	

**Oligonucleotides**		

mCherry_ApaI_f CCCCCCCCCGGGCCCTGAGCAGAAGCTGATCAGCGAGGAGGACCTGATGGTAAGCAAGGGCGAGGAGG	This paper	N/A
mCherry_ApaI_r CCCCCCCCCGGGCCCGCAGCAGCCTTGTACAGCTCGTCCATGCCGC	This paper	N/A
proIns-mChe_NheI_f1 CCCCCCCCCGCTAGCGCTACCGGACTCAGATCTCGAGCTCAAGCTTCCGCCATGGCCCTGTGGATGCG	This paper	N/A
proIns-mChe_BamHI_r CCCCCCCCCGGATCCAAGTTGCAGTAGTTCTCCAGCTGGTAGAGGG	This paper	N/A
sfGFP_BamHI_f CCCCCCCCCGGATCCGGATCCACCGGTC GCC ACC GGTGCAGGAGCTATGAGCAAGGGC	This paper	N/A
sfGFP_NotI_r GGTTTAAACGGGCCCTCTAGACTCGAGC	This paper	N/A
proins_R55S_mut1 CCAAGACCAGCCGGGAGGCAGAGGAC	This paper	N/A
proins_R55S_mut2 CCCGGCTGGTCTTGGGTGTGTAGAAGAAGC	This paper	N/A
proins_R94S_mut1 CTGCAGAAGAGTGGCATTGTGGAACAATGC	This paper	N/A
proins_R94S_mut2 CAATGCCACTCTTCTGCAGGGACCCCT	This paper	N/A

**Recombinant DNA**		

Human proinsulin	OriGene	
sfGFP		
mCherry		
EGFP-phogrin	Seiji Torii	
CMV-B-GECO1	Robert Campbell	Addgene #32448

**Software and Algorithms**		

Fiji	Schindelin et al., 2012	
FluoQ	Stein et al., 2013	
ggplot2	Wickham, 2009	
ImageJ	Schneider et al., 2012	
Pearson_calculation.ijm	This paper	https://github.com/fstein/pearson_calculation
SE_analysis.ijm	This paper	https://github.com/fstein/SE_analysis

### Contact for Reagent and Resource Sharing

Further information and requests for resources and reagents should be directed to the Lead Contact, Carsten Schultz (schultz@embl.de)

### Experimental Model and Subject Details

#### Cell Line

Mouse MIN6 cells (a kind gift from the Miyazaki laboratory, Osaka University) were cultured between passage number 25 and 35. β-Mercaptoethanol was added freshly to the medium consisting of DMEM (Lifetechnologies), 1% penicillin/streptomycin (Gibco), 15% Fetal Bovine Serum (FBS, Gibco). Cells were maintained under 8% CO_2_ at 37°C and subcultured as previously described(Ishihara et al., 1993, Nakashima et al., 2009, Miyazaki et al., 1990, Cheng et al., 2012). For microscopy experiments, cells were seeded on slides within eight-well Lab-Tek Chambered Coverglass systems (Thermo Scientific) at 60-80% confluency. MIN6 cells were transfected using Lipofectamine 2000 (Invitrogen) according to the manufacturer’s protocol. After 24 h the transfection medium was replaced by culture medium followed by another 24 h incubation period. For the experiments indicated, medium was exchanged prior to imaging and cells were allowed to adapt 30 min for to imaging buffer IB (115 mM NaCl, 1.2 mM CaCl_2_, 1.2 mM MgCl_2_, 1.2 mM K_2_HPO_4_, 0.2% glucose and 20 mM HEPES, pH 7.4). Tolbutamide (Sigma) was dissolved in DMSO and applied for 30 min at 100 μM (100 mM stock in DMSO).

#### Culture of Primary Mouse β-Cells

Islets were isolated from C57BL6 mice by collagenase digestion, as previously described [J Frank et al Nat Chem Biol 2016]. Briefly, mice were euthanized by cervical dislocation and the bile duct was injected with a collagenase solution (1 mg/mL) before digestion at 37 °C for 10 min and separation of islets using a Histopaque (Sigma-Aldrich) gradient (1.083 and 1.077 g/mL). Islets were cultured for 24–72 h in RPMI medium supplemented with 10% FCS, 100 U/mL penicillin and 100 μg/mL streptomycin. Islets were dissociated into single β-cells using trypsin digestion for 5 min at 37 °C and allowed to attach to poly-L-lysine-coated and acid-etched coverslips. Animals were housed in the European Molecular Biology Laboratory (EMBL) animal facilities under veterinarian supervision and the guidelines of the European Commission, revised directive 2010/63/EU, and AVMA guidelines 2007.

### Method Details

#### Experimental Procedures

##### Gene Construction and Reagents

The mCherry cDNA was cloned in a human proinsulin vector (OriGene) between the insulin B and A chains using ApaI restriction enzyme (ThermoScientific), similar to the Emerald tagging strategy by Watkins (Watkins et al., 2002). After subcloning RINS1_mCherryonly_ using NheI/BamHI, the superfolder sfGFP was fused to the C-terminus of the insulin A chain using BamHI and NotI. RINS1mut was generated by mutation of amino acids 55 and 94 by mutagenesis PCR using the primers listed (Key Resources Table). EGFP-phogrin (Torii et al., 2004) was kindly provided by Seiji Torii (Gunma University) and CMV-B-GECO1 was a gift from Robert Campbell (Addgene plasmid #32448) (Zhao et al., 2011).

##### Western Blot Analysis

MIN6 cells were split and grown in 10 cm dishes to a confluency of 60-80%. After 24h they were transfected with RINS1wt or RINS1mut using Lipofectamine 2000 and cultured for 30 h. Cells were harvested using a cell scraper and lysed in lysis buffer (10 mMTris/Cl pH 7.5, 150 mM NaCl, 0.5 mM EDTA, 0.5% NP-40) supplemented with cOmplete (EDTA-free) protease inhibitor cocktail (Roche Diagnostics, Indianapolis, IN) and 1 mM PMSF (Sigma). Lysates (50 μg) were separated on NuPAGE 4-12% Bis-Tris protein gels (10-well, Thermo Scientific) and transferred to PVDF membranes (Immobilon-P, Millipore), blocked using 5% skim milk in PBS-T. The primary antibody anti-insulin, anti-GFP or anti-mCherry (Molecular Probes) was incubated in 5% skim milk overnight. The HRP-conjugated anti-rabbit secondary antibody (Zymed) was incubated for 30 min and imaged using a BioRad imaging system.

##### Confocal and TIRF Microscopy

Confocal microscopy was performed using a Zeiss LSM 780 NLO confocal microscope at 37°C, equipped with a 63 × 1.40 oil objective. Definite Focus (Carl Zeiss) was used to minimize focus shifts during time-lapse experiments. For excitation of sfGFP and mCherry 488 nm and 561 nm laser lines were used, respectively.

Time-lapse TIRF microscopy was performed at 37°C on an Olympus Biosystems CellˆTIRF system using an Olympus APON 100× oil TIRF objective (NA 1.49). To excite B-GECO1, sfGFP and mCherry, 405 nm, 488 nm and 561 nm solid state lasers were used, respectively. The laser alignment was performed individually for each region of interest. Image acquisition was operated by an xCELLence software package. The laser-based Z-drift compensator (ZDC) function was used to avoid focus shifts. Images were acquired every second for 4-5 min, with an exposure time of 100 ms.

##### Correlated Light and Electron Microscopy

Transiently transfected with the RINS1 sensor MIN6 cells were cryo-immobilized by high pressure freezing to preserve the native cellular ultrastructure. The cells were processed for electron microscopy (EM), thick-sectioned (300 nm) and labeled with fiducial markers (Tetraspecks) that can be seen by both fluorescence microscopy and electron tomography to correlate the images. RINS1 fluorescence appeared as a granular pattern with mostly overlapping mCherry and sfGFP signals similar to live cell confocal microscopy. The coordinates of the fluorescence signals were correlated to the electron tomogram with high precision based on the Tetraspeck coordinates. Fluorescent spots localized to discrete granular structures in the electron tomograms. In some cases we could achieve subgranular localization of sfGFP-insulin and C-peptide-mCherry demonstrating separation of both proteins within a granule. Due to the complex procedure, more details of this CLEM experiments will be presented in a future paper that is currently in preparation.

### Quantification and Statistical Analysis

#### FRET Calculation

Sensitized emission FRET data were acquired by confocal microscopy of MIN6 transfected by RINS1wt, RINS1mut, RINS_Cherryonly_ and RINS_sfGFPonly_ and fixed by 4% PFA. Z-stacks of sfGFP (donor D, donor excitation, donor emission), mCherry (acceptor A, acceptor excitation acceptor emission), FRET (FRET S, donor excitation, acceptor emission) channels were acquired. RINS_Cherryonly_ and RINS_sfGFPonly_ data were used to calculate the bleed-through coefficient β = S/D = 0.245 and the cross-excitation coefficient γ =S/A = 0.072. Sensitized emission (SE-FRET) for RINS1wt andRINS1mut was calculated according to the formula SE=S-β*D-γ*A(van Rheenen et al., 2004). Prior to the image analysis, all images were processed by applying a median filter (radius size = 1), converting them to 32-bit and using a threshold (sfGFP = 40, FRET = 10, mCherry = 40) to remove low value pixels. The image SE-FRET was calculated with our newly developed ImageJ macro SE_analysis.ijm (see Supplemental Information and Figure S7 for details). The macro computes the corrected FRET image SE-FRET and normalizes the channel with the donor D by calculating the SE-FRET-sfGFP-ratio. We calculated an average SE-FRET over all z-stacks and plotted the SE-FRET over sfGFP channel ratio as well as the donor and acceptor channels normalized to the respective mean RINS1wt value (see Figure S7).

#### Image Analysis

Images were analyzed using Fiji (Schindelin et al., 2012) and FluoQ (Stein et al., 2013). For the analysis of ratiometric time-lapse image data, the following options were chosen in the initial graphical user interface of FluoQ: No background or smoothening methods was chosen, images were thresholded automatically with the ‘Triangle’ method and cells were segmented manually. mCherry was used as the denominator and sfGFP as the numerator channel. The output file of FluoQ named $Experiment.name_dataset.txt was subsequently loaded into R(Team, 2011) and results were plotted using the ggplot2 (Wickham, 2009) package.

Since FluoQ is capable of analyzing time-series data only, the Z-stack of images with no time-correlation was transformed into the time dimension with the following short ImageJ(Schneider et al., 2012) macro:

run(“Bio-Formats Importer”, “open=[C:\\Path_to_file\\example.lsm]”);
Stack.getDimensions(width, height, channels, slices, frames);
Stack.setDimensions(channels,frames,slices);
saveAs(“Tiff”, “C:\\Path_to_file\\example.tif”);

The resulting tif images could be subsequently analyzed by FluoQ.

##### Colocalization (Pearson) Analysis

The Pearson colocalization coefficient between two channels was calculated using our newly developed ImageJ macro Pearson_calculation.ijm (see Figure S6 for details). The macro computes the Pearson coefficient separately for each cell ROI of an image. Presumably, the Pearson value can be measured for the same cell over time or at different slices of a Z-stack. For the analysis, images were loaded into FIJI or ImageJ and cells were segmented. After background subtraction, a threshold was applied and zero value pixels were excluded from the subsequent analysis. After starting the Pearson_calculation macro, the Pearson value is calculated automatically as shown in Figure S6.

### Data and Software Availability

The custom scripts Pearson_calculation.ijm macro (ImageJ) (Figure S6) and SE_analysis.ijm macro (ImageJ) (Figure S7) have been deposited (Key Resources Table link).

## Author Contributions

C.S., M.S., and D.A.Y. designed the study. M.S., D.A.Y., and A.B. performed the experiments, F.S. performed image analysis. All authors contributed to the manuscript writing.

## Supplemental Information

Document S1. Figures S1–S14 and Table S1

Movie S1. Merged Images Display sfGFP in Green and mCherry in Magenta Acquired by TIRF Microscopy; 20 mM Glucose Was Added after 60 s and the Time-Lapse Images Were Taken Every Second for 240 s

Movie S2. The B-GECO Emission Intensity on the Right, Fire, as well as RINS1 with mCherry in Red and sfGFP in Green, Recorded for 300 s

Document S2. Article plus Supplemental Information
