# Supplementary material for: A Ratiometric Sensor for Imaging Insulin Secretion in Single β Cells
Source: Cell Chem Biol. 2017 Apr 20;24(4):525–531.e4. doi: 10.1016/j.chembiol.2017.03.001 (PMC5404835; doi:10.1016/j.chembiol.2017.03.001)
Supplement: Document S1. Figures S1–S14 and Table S1 [file mmc1.pdf]

Cell Chemical Biology, Volume *24*

Supplemental Information

A Ratiometric Sensor for Imaging Insulin Secretion in Single β Cells

Martina Schifferer, Dmytro A. Yushchenko, Frank Stein, Andrey Bolbat, and Carsten Schultz

Supporting Information

A ratiometric sensor for imaging insulin secretion in single β-cells

Supplementary Figures

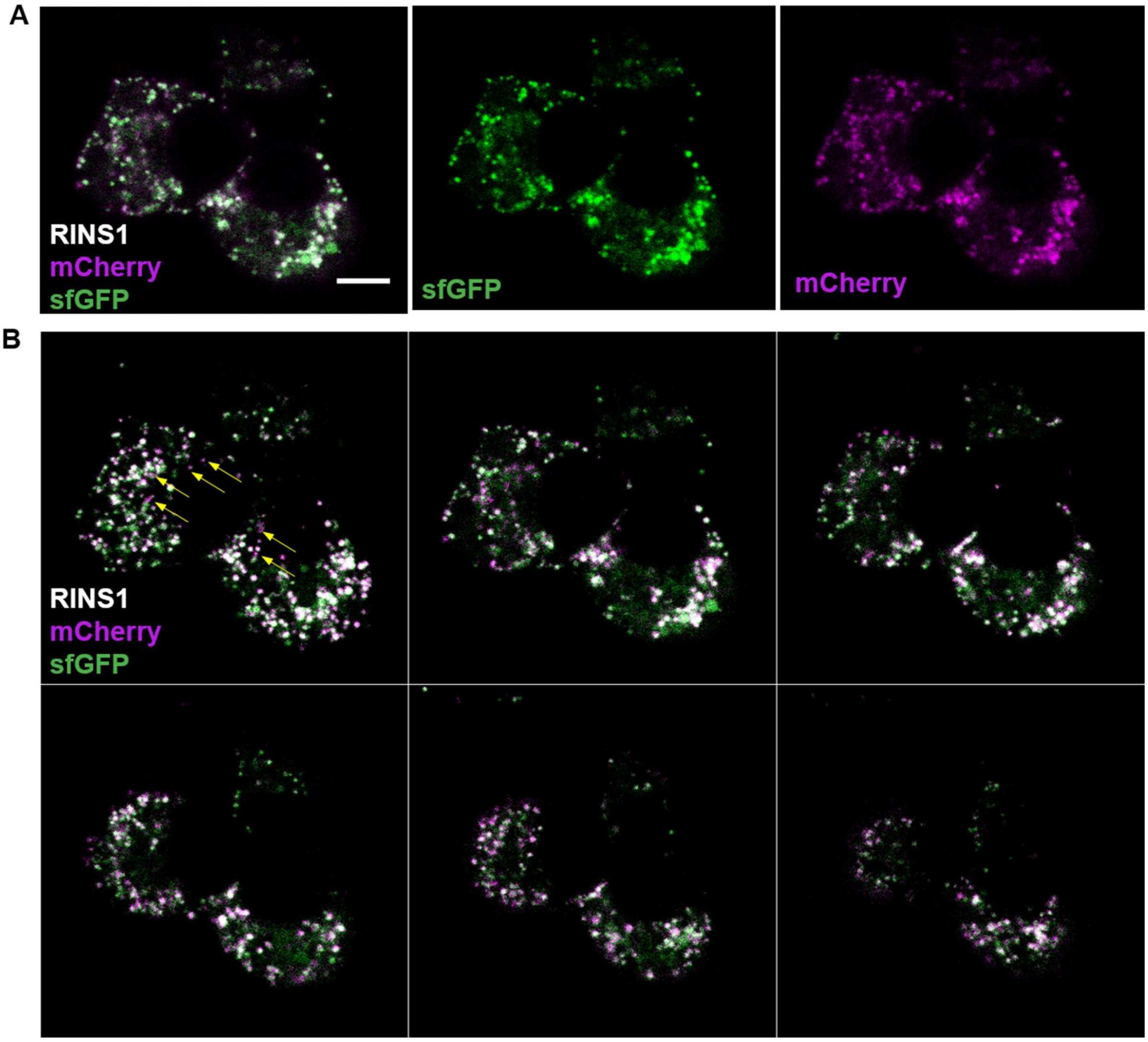

Figure S1. Expression of RINS1 in MIN6. Confocal microscopy of RINS1 in MIN6 cells. (A) RINS1 emission in single (middle, right) and merged (left) channels (sfGFP, green; mCherry, magenta) Scale bar 5 µm (B) Different confocal z-planes of the 3 cells from (A). Most granules close to the cell culture dish (top left) show emission from both fluorescent proteins with some being positive for mCherry only (yellow arrows).

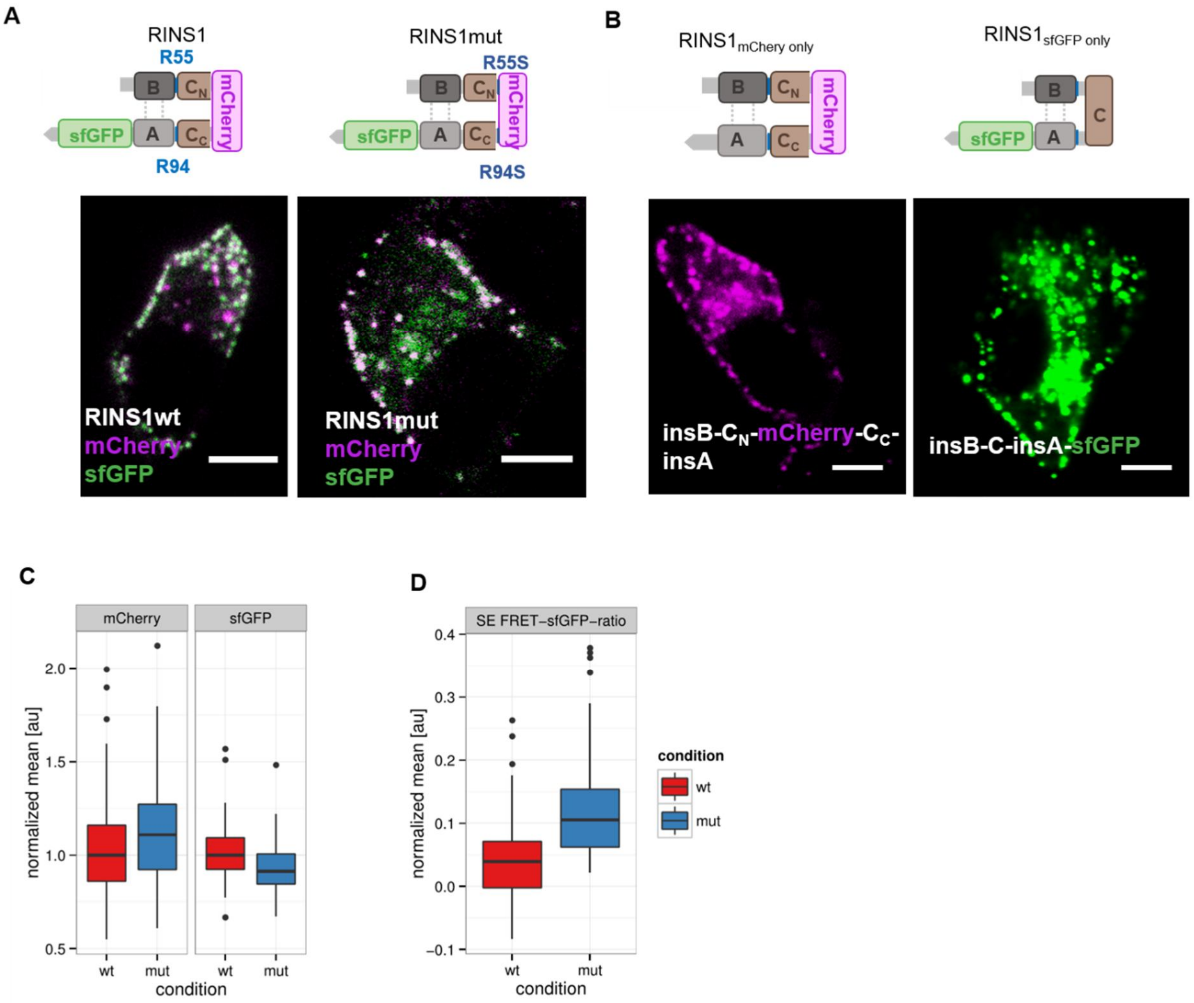

Figure S2. FRET measurements in RINS1 transfected MIN6. (A-B) Confocal imaging of MIN6 cells expressing the indicated proinsulin constructs used for the calculation of coefficients to correct for bleed-through and cross-excitation. Fixed MIN6 cells transfected by (A) RINS1 (left) and RINS1mut (right) merged channels (sfGFP, green; mCherry, magenta) (B) MIN6 cells expressing RINS1$_{Cherryonly}$ (mCherry, magenta) or RINS1$_{sfGFPonly}$ (sfGFP, green), respectively. Merged images of both channels are shown. Scale bar 5 µm. (C) MIN6 cells were transfected with RINS1 (red, n=63) and RINS1mut (blue, n=61), respectively. After 1 day, we imaged mCherry (acceptor A), sfGFP (donor D) and FRET (S) channels of z-stacks by confocal microscopy. mCherry and sfGFP were normalized and plotted (D). We determined FRET as sensitized emission (SE) by calculation of FRET coefficients β and γ as well as the SE-FRET (SE) according to the formula (SE=S-β*D-γ*A). The ratio of SE-FRET to sfGFP was plotted for RINS1 and RINS1mut transfected MIN6 cells.

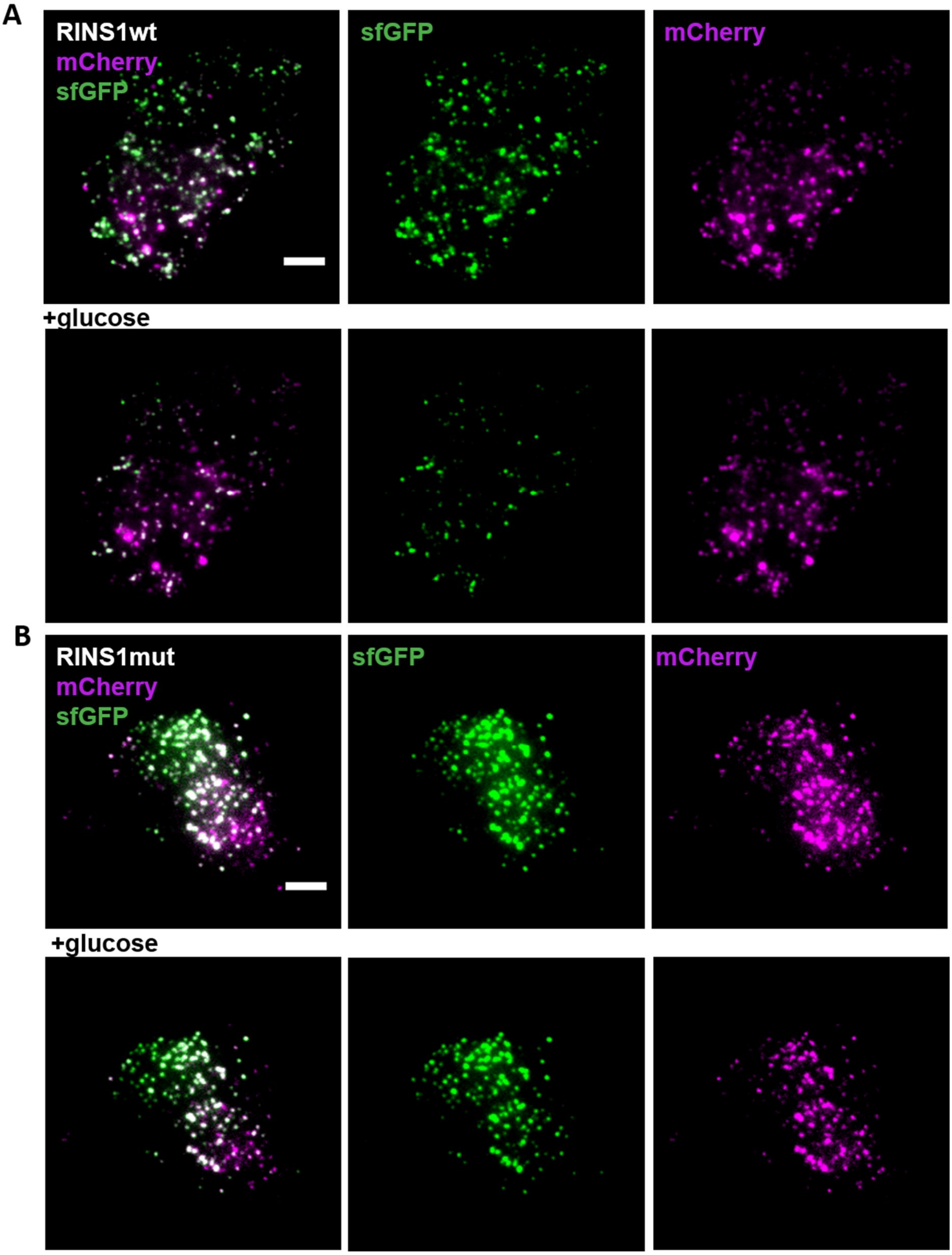

Figure S3. RINS1 and RINS1mut TIRF microscopy. MIN6 cells expressing RINS1 (A) and RINS1mut (B) for 48 h were imaged before (top) and after (bottom) glucose stimulation. Merged (left) and single channels for sfGFP (green, middle) or mCherry (magenta, right) are shown. Scale bar 5 µm.

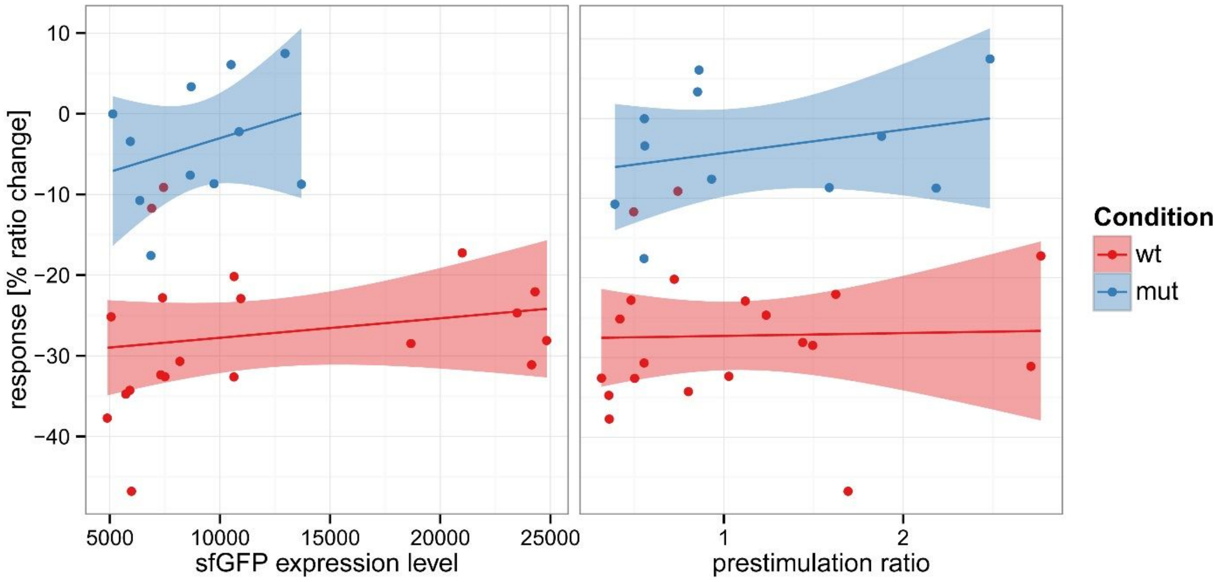

Figure S4. Correlation of RINS1 expression levels and its ratio response amplitude. MIN6 cells expressing RINS1 (red, n=18) or RINS1mut (blue, n=11) were imaged by TIRFM. After 60 sec 20 mM glucose was added. The maximal sfGFP/mCherry emission ratio drop upon stimulation was plotted against the sfGFP emission intensity (AU) (left) and the sfGFP/mCherry emission ratio at time point zero before glucose stimulation. Plots show trend lines including the confidence intervals and points representing individual cells.

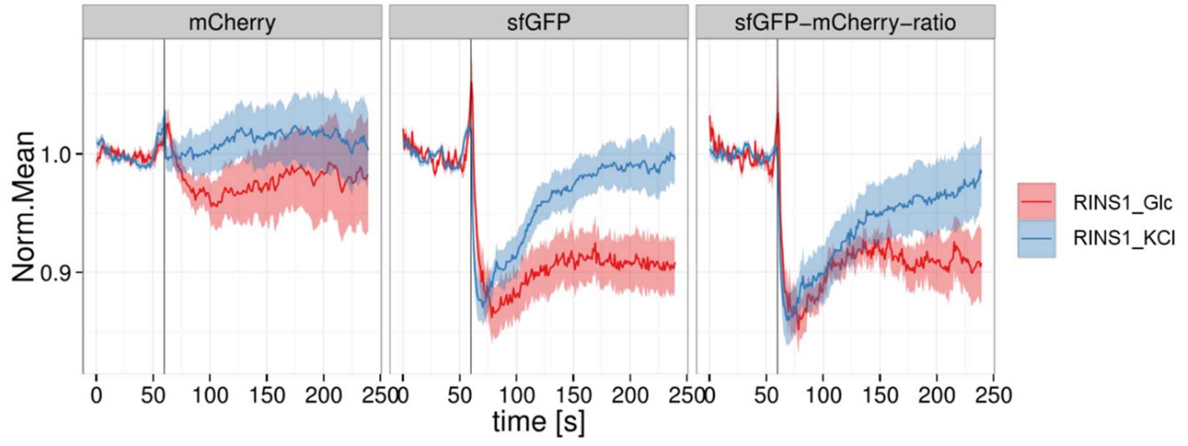

Figure S5. Comparison of different stimuli for insulin secretion. MIN6 cells expressing RINS1 were imaged by TIRFM. After 60 sec, either 20 mM glucose (red, n=20) or 20 mM KCl (blue, n=18) were added. Emission intensities for both FPs and their ratio (sfGFP/mCherry) were quantified.

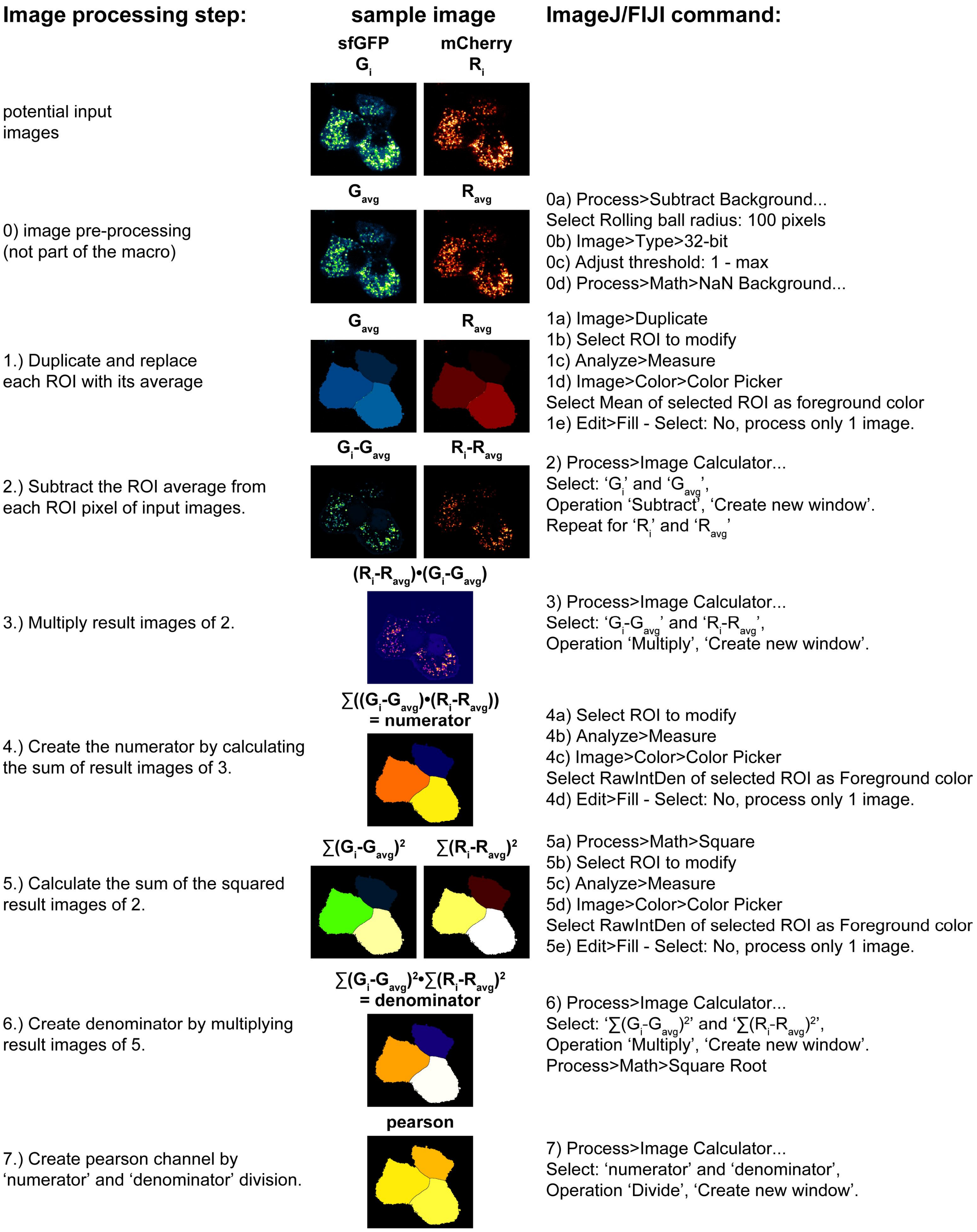

Figure S6. Image processing pipeline of the Pearson_calculation.ijm macro (ImageJ) calculates the Pearson colocalization coefficient for each ROI between two images. It also works for time-series or Z-stack data. The result is an image in which each pixel of a ROI has the value of the Pearson coefficient. The macro can be started once the two channels are loaded into FIJI or ImageJ, cells are segmented and images are preprocessed as indicated in step 0.

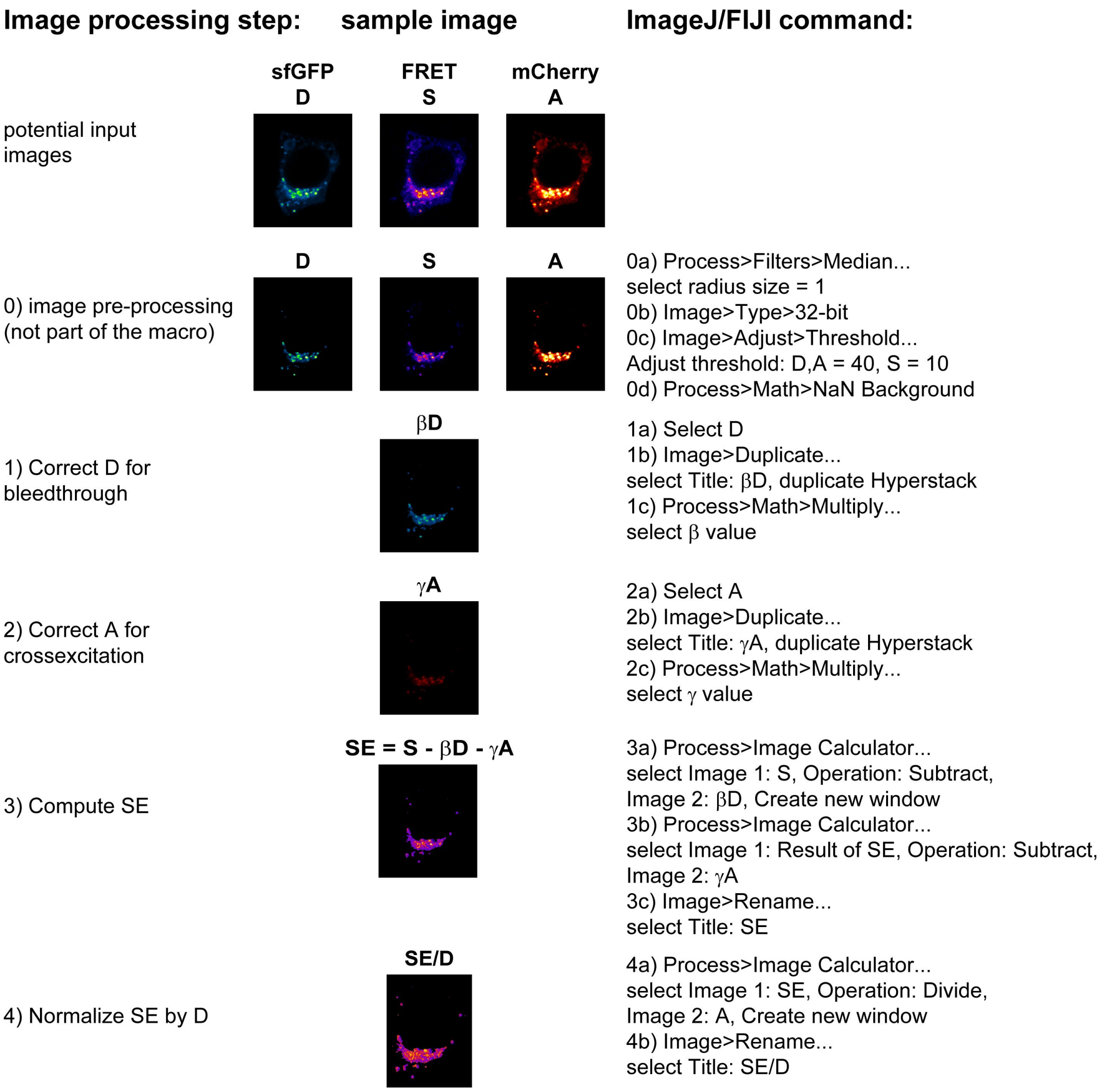

Figure S7. Image processing pipeline of SE_analysis.ijm macro (ImageJ) corrects a FRET image S (donor excitation, acceptor emission) for bleed-through of the donor D (donor excitation and donor emission) and cross-excitation of the acceptor A (acceptor excitation and acceptor emission). The macro can be started once the three channels D, S, and A are loaded into ImageJ or FIJI. Cells are segmented and images are preprocessed as indicated in step 0.

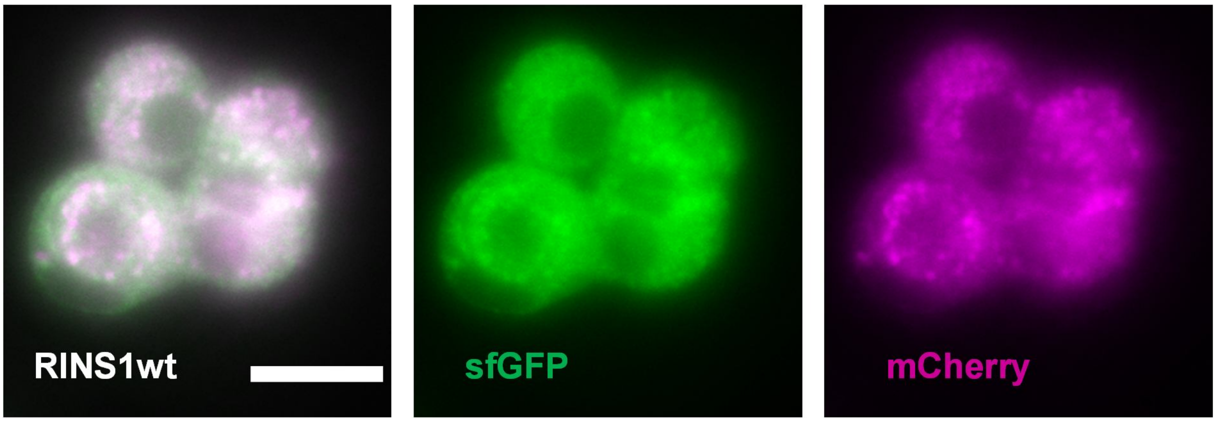

Figure S8. Expression of RINS1 in primary murine β-cells. Wide-field microscopy of RINS1 in primary β-cells obtained from the dissociation of islets of Langerhans and transduced with

adenoviral vector to deliver RINS1 DNA into cells (after 30 h of transduction). The adenoviral vector (Type 5, dE1/E3) containing the RINS1 transgene for expression under the control of CMV promoter was developed and prepared by Vector Biolabs. For transduction of primary cells, a $5.9x10^7$ PFU/ml titer was used. Imaging was performed in imaging buffer with no glucose, scale bar 10 µm.

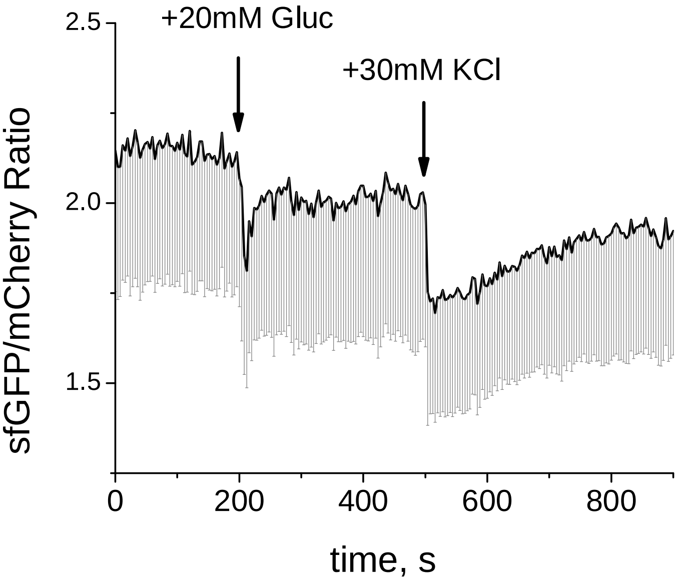

Figure S9. Monitoring stimulated insulin secretion in primary β-cells. Quantification of TIRF imaging data of primary β-cells cells expressing RINS1 (8 cells from 3 experiments) stimulated after 200 seconds with 20 mM glucose and following stimulation with 30 mM KCl at 500s.

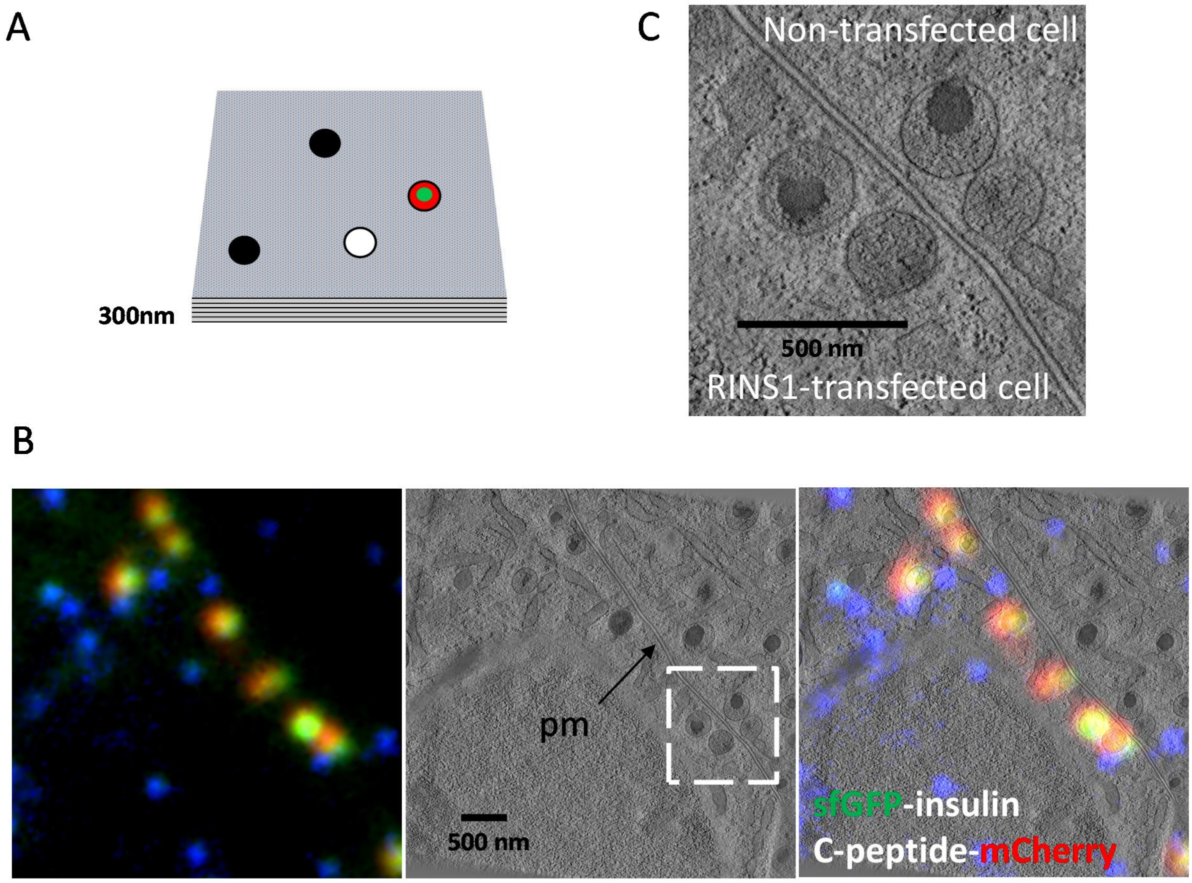

Figure S10. High-precision correlative fluorescence and electron microscopy (CLEM) of MIN6 cells. A) Schematic representation of electron tomography sections indicating the electron microscopy image location within the 300 nm thick section. Blue - fiducial markers Tetraspecks; green-red – granules of RINS1 transfected cells with fluorescent proteins (sfGFP-insulin and C-peptide-mCherry); black and white - granules of not transfected cells with and without insulin respectively. B) Fluorescence image (left, scale bar 500 nm) of two MIN6 cells, 300 nm section with fiducial markers (blue). One plane of a tomogram of the same cell (middle, scale bar 500 nm) and the correlated image (right). Note the plasma membranes are dividing a transfected (RINS1+) and a non-transfected (nt) cell, pm - plasma membrane. C) Enlargement of the selected region from the image B demonstrating the similarity in morphology of granules from RINS1 expressing cells and non-transfected cells. Both types of cells have granules with characteristic crystalline structure of insulin (regions with high electron density on the image).

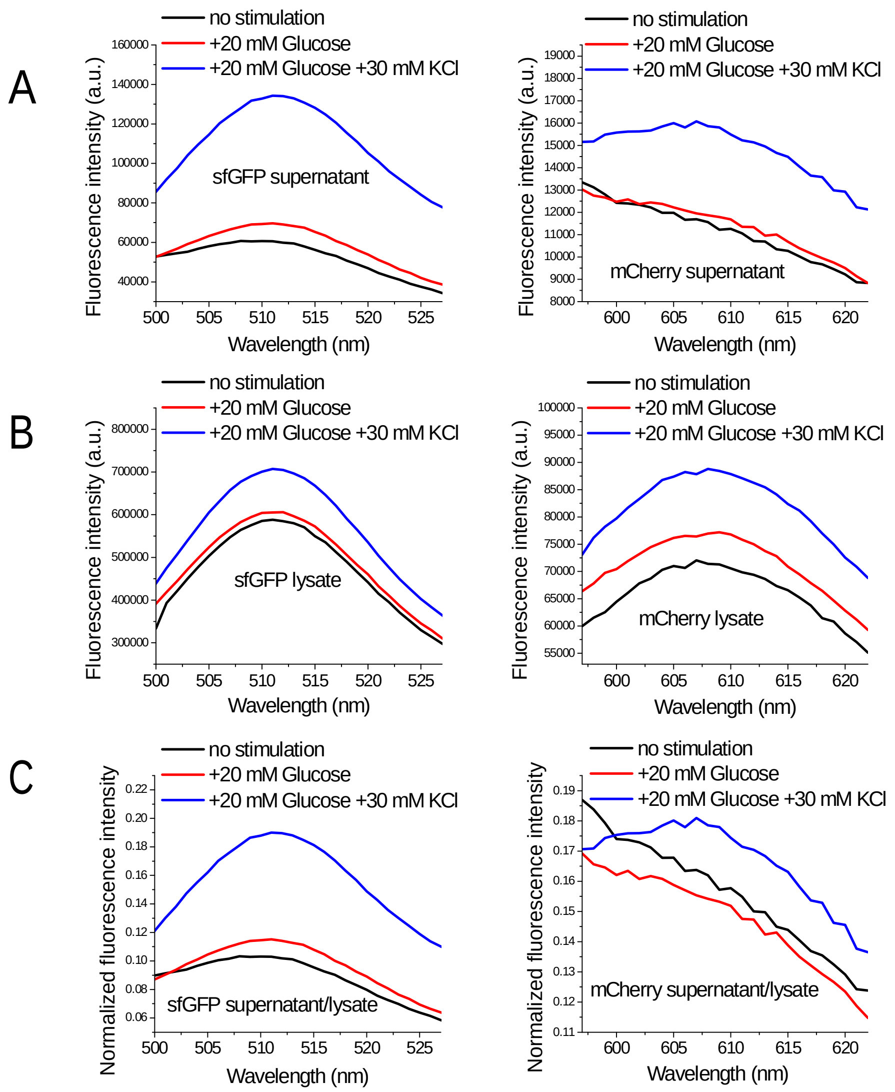

Figure S11. Emission spectra of RINS1. Comparison of A) fluorescence emission spectra of secreted sfGFP and mCherry fusion proteins, B) fluorescent spectra of sfGFP and mCherry fusion proteins from lysates, and C) fluorescence intensity of secreted sfGFP (λ_{ex} = 511 nm) and mCherry (λ_{ex} = 608 nm) fusion proteins normalized to the fluorescence of these proteins in lysates of MIN6 cells transiently expressing RINS1 sensor. MIN6 cells in 6 cm dishes were incubated either with no glucose (control), 20 mM glucose or 20 mM glucose + 30 mM KCl in 1ml of imaging buffer for 1 h. Afterwards the supernatant was removed (and later used for fluorescence measurements) and cells were lysed (lysis buffer: 10 mM Tris/Cl pH 7.5, 150 mM NaCl, 0.5 mM EDTA, 0.5% NP-40 supplemented with cOmplete protease inhibitor cocktail and 1 mM PMSF). Lysates were resuspended in 1 ml of imaging buffer and their fluorescence properties were investigated.

Table S1. Comparison of sfGFP/mCherry fluorescence intensity ratios as described in the Fig S11.

sfGFP/mCherry intensity ratios of secreted RINS1:	
no stimulation	0.65
+20 mM Glucose	0.75
+20 mM Glucose +30 mM KCl	1.08

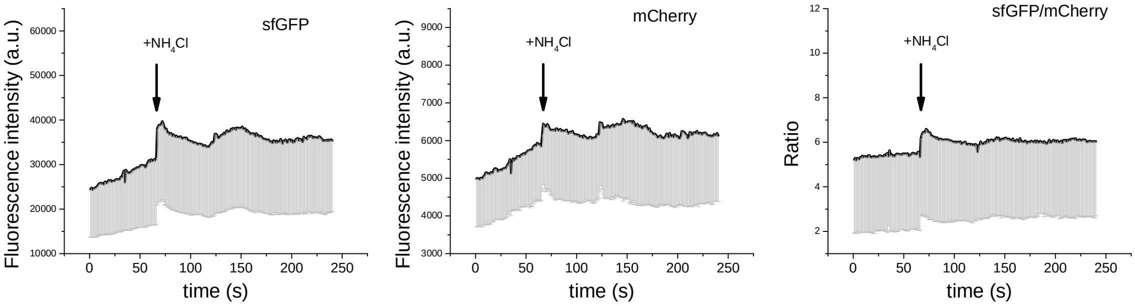

Figure S12. pH-sensitivity of RINS1 sensor. Response of MIN6 cells expressing RINS1 to the addition of 20 mM NH_4Cl at 65 s during TIRF imaging (7 cells in 2 experiments). There is an increase in emission of sfGFP and almost no change of the mCherry signal. This result excludes a contribution of pH to the observed decrease of the sfGFP/mCherry ratio during secretion. In fact, any contribution of decreasing pH during secretion would be counteracting the observed sfGFP/mCherry ratio change.

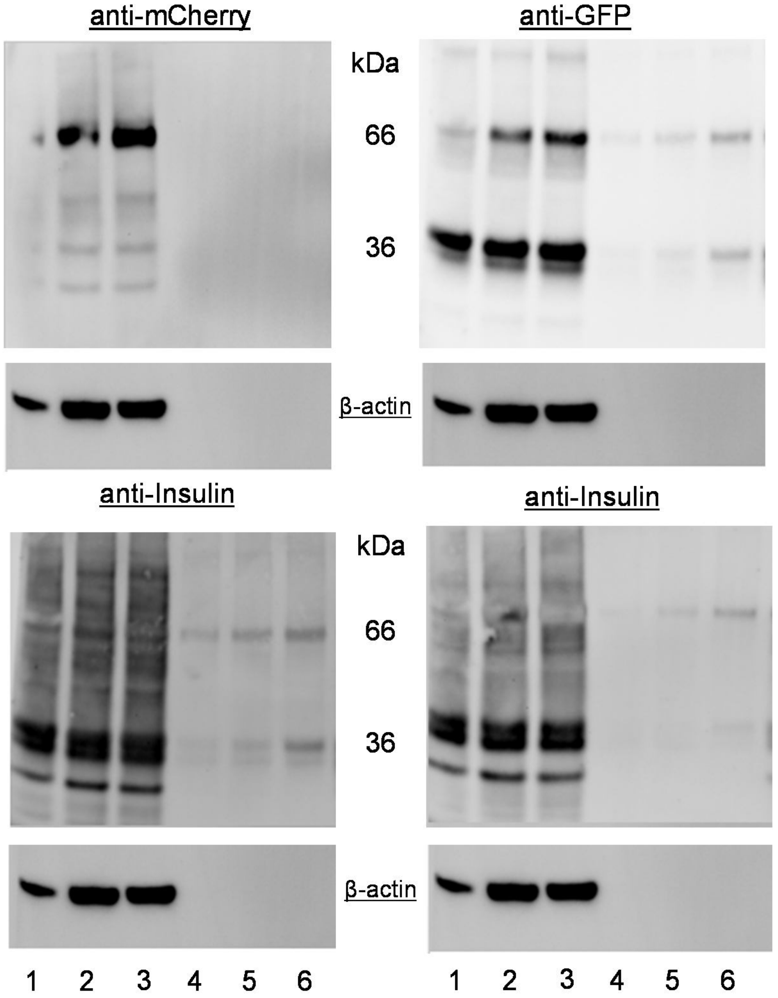

Figure S13. Western blot analysis with anti-mCherry, anti-GFP and anti-insulin antibodies of the secreted proteins and cellular content from MIN6 cells transiently expressing RINS1 sensor.

Lanes:

1 - lysate of the cells incubated in the imaging medium in the absence of glucose;

2 - lysate of the cells incubated for 1h in 20 mM glucose;

3 – lysate of the cells incubated for 1h in 20 mM glucose and 30 mM KCl;

4 - supernatants from the same cells that were incubated in the imaging medium in the absence of glucose;

5 – supernatants from the same cells that were incubated for 1h in 20 mM glucose;

6 – supernatants from the same cells that were incubated for 1h in 20 mM glucose and 30 mM KCl.

These data indicate that C-peptide-mCherry forms dimers in secretory vesicles that is not observed for sfGFP-insulin.

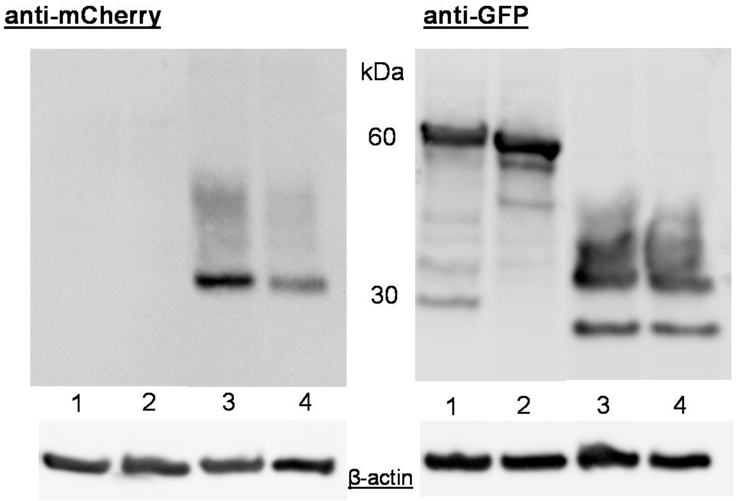

Figure S14. Determination of specificity of the anti-GFP and anti-mCherry antibodies used for Western blots. Lane **1** - EGFP-A4; **2** - A4-EGFP; **3, 4** – mCherry.

Supplemental Movies

Movies for MIN6 cells transfected with RINS1 and treated for 30 min with tolbutamide (100 µM) are attached.

Movie 1. The merged images in Movie 1 "RINS1_tolbutamide" display sfGFP (green) and mCherry (magenta) acquired by TIRF microscopy. Glucose (20 mM) was added after 60 seconds. The time lapse images were taken every second for 240 seconds.

Movie 2 "RINS1_BGECO_tolbutamide" additionally shows the B-GECO emission intensity (right, fire) as well as RINS1 (mCherry, red; sfGFP, green) and was recorded for 300 sec.
