# Supplementary figures and images for: A Ratiometric Sensor for Imaging Insulin Secretion in Single β Cells
Source: Cell Chem Biol. 2017 Apr 20;24(4):525–531.e4. doi: 10.1016/j.chembiol.2017.03.001 (PMC5404835; doi:10.1016/j.chembiol.2017.03.001)

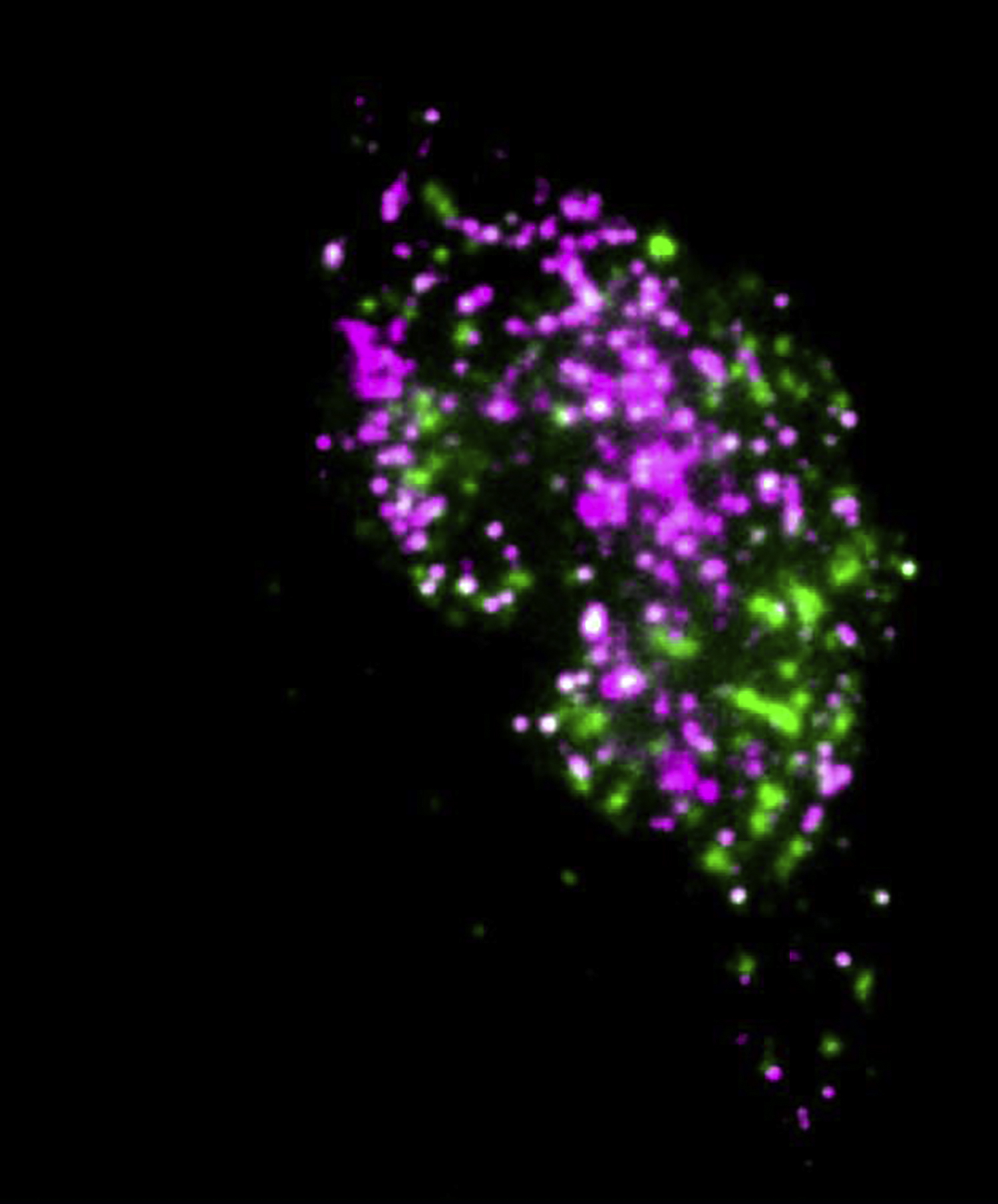

Supplement: Movie S1. Merged Images Display sfGFP in Green and mCherry in Magenta Acquired by TIRF Microscopy; 20 mM Glucose Was Added after 60 s and the Time-Lapse Images Were Taken Every Second for 240 — s [file mmc2.jpg]

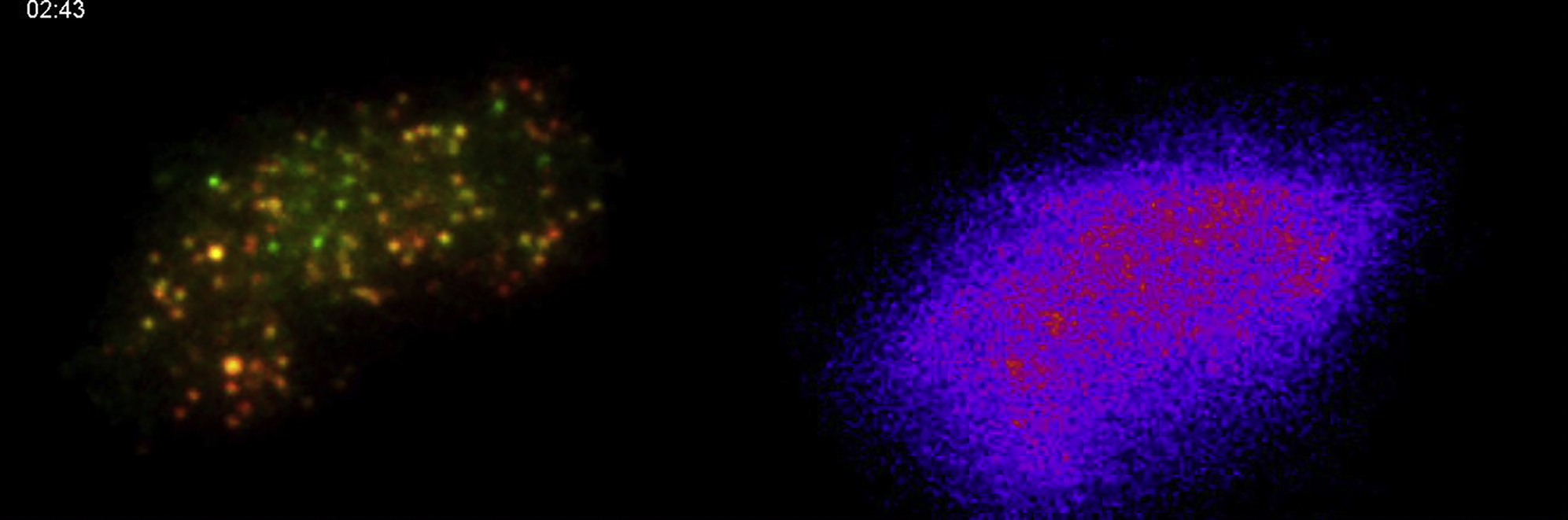

Supplement: Movie S2. The B-GECO Emission Intensity on the Right, Fire, as well as RINS1 with mCherry in Red and sfGFP in Green, Recorded for 300 — s [file mmc3.jpg]
